# Predominance of *Trichophyton tonsurans* causing tinea capitis: A 12-year retrospective study in the north of Iran

**DOI:** 10.18502/CMM.2023.345026.1395

**Published:** 2023-03

**Authors:** Mohammad T. Hedayati, Firoozeh Kermani, Javad Javidnia, Mahmood Moosazadeh, Mohsen Nosratabadi, Maryam Salimi, Sabrieh Asadi, Elham Mosayebi, Zohreh Hajheydari, Masoud Golpour, Ghasem Rahmatpour Rokni, Armaghan Kazemi Nejad, Tahereh Shokohi, Felix Bongomin

**Affiliations:** 1 Invasive Fungi Research Centre (IFRC), Communicable Diseases Institute, Mazandaran University of Medical Sciences, Sari, Iran; 2 Department of Medical Mycology, School of Medicine, Mazandaran University of Medical Sciences, Sari, Iran; 3 Health Science Research Center, Addiction Institute, Mazandaran University of Medical Sciences, Sari, Iran; 4 Student Research Committee, School of Medicine, Mazandaran University of Medical Sciences, Sari, Iran; 5 Department of Dermatology, School of Medicine, Bu-Ali Sina Hospital, Mazandaran University of Medical Sciences, Sari, Iran; 6 Department of Medical Microbiology and Immunology, Faculty of Medicine, Gulu University, Gulu, Uganda

**Keywords:** Iran, Prevalence, Tinea capitis, *Trichophyton tonsurans*

## Abstract

**Background and Purpose:**

Among different clinical entities of dermatophytosis, tinea capitis (TC) is considered a major public health challenge in the world, especially in regions with poor health and low income. Therefore, this study aimed to provide a retrospective analysis of the patients suspected of TC who were referred to the medical mycology laboratory of Mazandaran, a northern province of Iran.

**Materials and Methods:**

A retrospective analysis was performed on the patients suspected of TC who were referred to the medical mycology laboratory from July 2009 to April 2022. Hair roots and skin scrapings were collected from the participants. The laboratory diagnosis was confirmed by direct microscopic examination and culture. Finally, 921 out of 11095 (8.3%) patients were suspected of TC.

**Results:**

Based on the findings, TC was confirmed in 209 out of 921 patients (22.7%). In terms of gender, 209 TC patients (75.1%) were male. Moreover, the male to female ratio of TC patients was 1:3.0. *Trichophyton tonsurans* (146/174, 83.91%) was the most etiological agent, followed by *T. mentagrophytes* (13/174, 7.47%), *T. violaceum* (9/174, 5.17%), *Microsporum canis* (3/174, 1.71%), *T. verrucosum* (2/174, 1.15%) and *T. rubrum* (1/174, 0.57%). Besides, endothrix (77.0%) was the most prevalent type of hair invasion.

**Conclusion:**

The results revealed the predominance of *T. tonsurans*, as a causative agent of TC. Despite the prevalence of TC, the absence of appropriate consideration highlights that it is a neglected complication among children.

## Introduction

Tinea capitis (TC) is one of the most frequent, communicable, superﬁcial fungal infections with worldwide distribution that affects the scalp and hair [ 1 ]. Among different clinical entities of dermatophytosis, TC has remained a major public health challenge in the world, especially the regions with poor health and low income [ 2 ]. A previous review showed that TC is present in about 20% of the general population in developing countries [ 3 ].

Tinea capitis is usually a noninvasive and curable complication; however, its widespread nature and therapeutic costs are major public health concerns, imposing a high economic burden on society, especially in developing countries, including Iran. Treatment of dermatophyte infections incurs a significant cost burden. It has been estimated that over 500 million USD per year is spent worldwide on drugs to treat dermatophytosis [ 3 ]. In the United States, approximately the estimated economic burden of dermatophytosis was $1.2 billion in 2019 [ 4 ]. In another study conducted in the US, the average annual cost of TC treatment was estimated at $253 per patient, consisting of $158 for prescription drugs and $95 for medical services [ 5 ].

Overall, the severity of the disease depends on the interaction between the host and the etiologic agents and can lead to hair loss, scaling, erythema, and lesions that mimic impetigo, folliculitis decalvans, or scalp cellulitis [ 6 ]. Clinical features also vary according to the species of dermatophyte and type of hair invasion, ectothrix, endothrix, and favus [ 7 - 9 ]. The infection occurs mostly among school-aged boys and prepubescent children [ 10 ] and rarely in adults [ 11 ]. In Iran, TC is still regarded as a major public health. Recent observations have shown a gradual decline in the frequency of TC in Iran due to the improvement in sanitary and socio-economic conditions, but signiﬁcant changes have occurred in the dermatophytes responsible for infection [ 12 , 13 ]. 

Recently, the classification of the dermatophytes was significantly changed, and seven accepted genera were defined, namely *Trichophyton*, *Epidermophyton*, *Nannizzia*, *Paraphyton*, *Lophophyton*, *Microsporum*, and *Arthroderma* [ 14 ]. Among these, *Microsporum* and *Trichophyton* are the usual causative agents of TC [ 15 - 17 ]. However, the distribution of causative agents is inﬂuenced by a wide range of factors, such as gender, age, climate, type of population, lifestyle, migration of people, overcrowding, changing socio-economic status of the regions, immunity of the host, and antifungal therapy [ 18 - 20 ]. 

Nowadays, anthropophilic dermatophytes, in particular, *T. tonsurans* and *T. violaceum*, have emerged as the dominant agents in many regions in Iran [ 21 , 22 ]. A clear understanding of the epidemiology of TC will be useful in the development of preventive strategies and will help to increase the health awareness of the community. Therefore, the present study aimed to provide a retrospective analysis of the patients suspected of TC who were referred to the medical mycology laboratory of Mazandaran, a northern province of Iran from July 2009 to April 2022.

## Materials and Methods

This retrospective study analyzed the data of patients suspected of TC who were referred to the reference laboratory of medical mycology of Mazandaran, a northern province of Iran from July 2009 to April 2022. Demographic characteristics of patients, including age, gender, occupation, admission year, and the results of direct microscopic examination and culture of samples were extracted using a standardized data collection tool. 

Regarding sample collection during the study period, hair root and skin scraping samples were collected from the referred patients that were clinically suspected to have TC. The laboratory diagnosis of the aetiologic agent of TC was confirmed by direct microscopic examination using 20% potassium hydroxide and culture on Sabouraud dextrose agar (E. Merck, Germany) with cycloheximide and chloramphenicol. The plates were incubated for 2-4 weeks at 30 °C and checked weekly. Microscopically, cultured samples were evaluated by mode of formation, vegetative growth, number, shape, and arrangement of micro and macroconidia, as well as the appearance of chlamydospores [ 23 ]. 

Statistical analyses were performed in SPSS software (IBM version 18). Data were evaluated using Graph Pad Prism 5 Software and significant differences were determined using the chi-square test. It should be mentioned that P-values of less than 0.05 were considered statistically significant.

## Results

Overall, from July 2009 to April 2022, 921 (8.3%) out of 11095 referred patients to the laboratory were suspected to have TC. Tinea capitis was confirmed in 209 (22.7%) patients, 157/209 (75.1%) of whom were male. The rate of TC was 209/11095 (1.9%) among all referred patients suspected of having a type of fungal infection of skin, hair, and nails. The male-to-female ratio in suspected and confirmed patients with TC was 1:1.5 and 1:3.0, respectively. The statistical analyses showed a significant difference between patients with TC in terms of gender ‍‍‍‍‍‍(χ 2=25.27, *P<0.0001*). 

**Figure 1:**
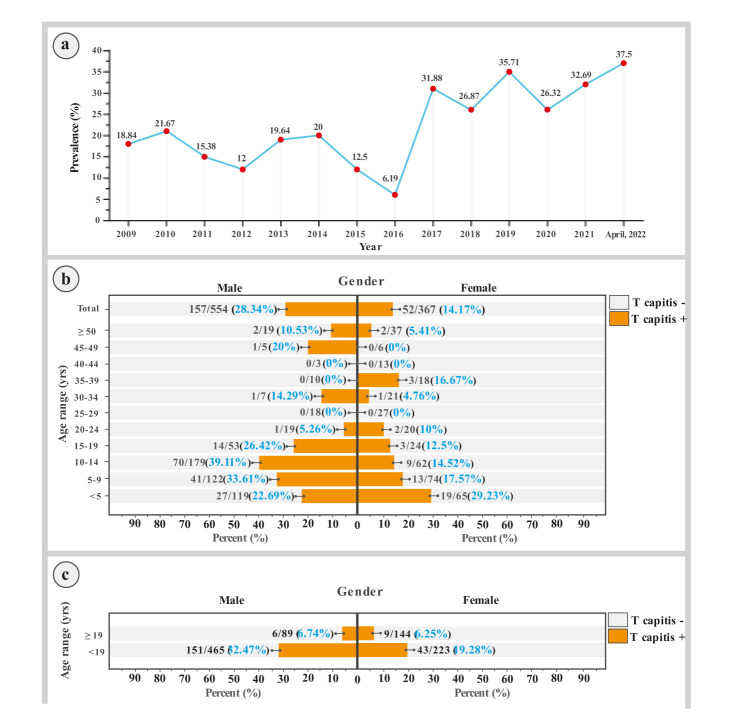
(a): Frequency distribution of dermatophytes with respect to years, (b): Dermatophytes isolation with respect to gender and age groups and (c): Comparison of the frequency distribution of tinea capitis in two age groups of < 19 years old and ≥ 19 years old

During the study, the rate of TC did not follow a regular pattern based on the year of study. It decreased from 2010 to 2012, increased from 2012 to 2014, decreased from 2014 to 2016, and increased from 2016 until April 2022 fluctuating between increases and decreases (Figure 1a). The lowest and highest calculated frequencies of TC were observed in 2016 (6.2%) and 2019 (35.7%), respectively (Figure 1a).

The average age of suspected patients was 16.0±15.0 years old (range: 0-91 years old), and the mean age of patients with TC was 10.6±8.7 years old. Patients affected with TC (37.8%) were mainly within the age range of 10-14 years old, followed by the age range of 5-9 (25.8%) and below five years old (22.0%). Most patients with TC, in both genders, were younger than 19 years old (Figure 1b, c). Moreover, it should be mentioned that most patients with TC were wrestlers (68.9%).

**Figure 2:**
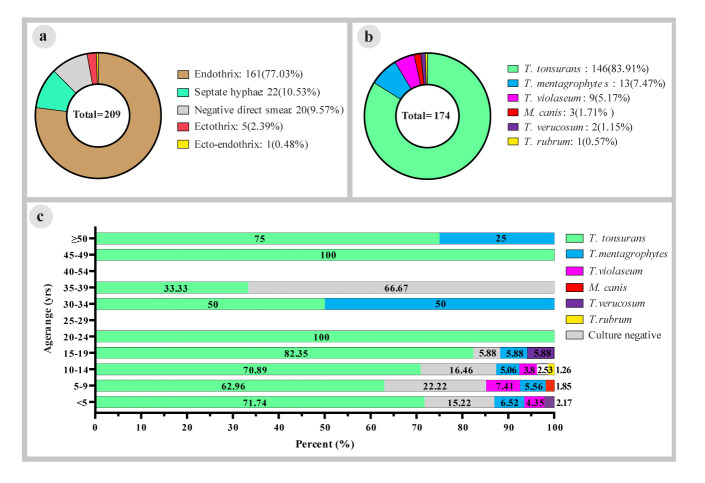
(a): Frequency of hair invasion classification of tinea capitis based on direct examination, (b): Frequency distribution of various agents among confirmed dermatophytes, (c): Dermatophyte species diversity according to age groups

Regarding the type of hair invasion, endothrix, ectothrix, and ecto-endothrix forms were observed in 161/209 (77.03%), 5/209 (2.39%), and 1/209 (0.48%) cases, respectively. In 22/209 cases (10.53%), septate hyphae were also observed in collected crust samples from the scalp (Figure 2a). The most common causative agent of endothrix form was *T. tonsurans* 116/161 (72.04%) followed by *T. violaceum* 8/161 (4.5%) and *T. mentagrophytes* 6/161 (3.72%). *Microsporum canis* and *T. mentagrophytes* (2/5, 40.0%, each) were identified as the causative agents of ectothrix form.

**Figure 3:**
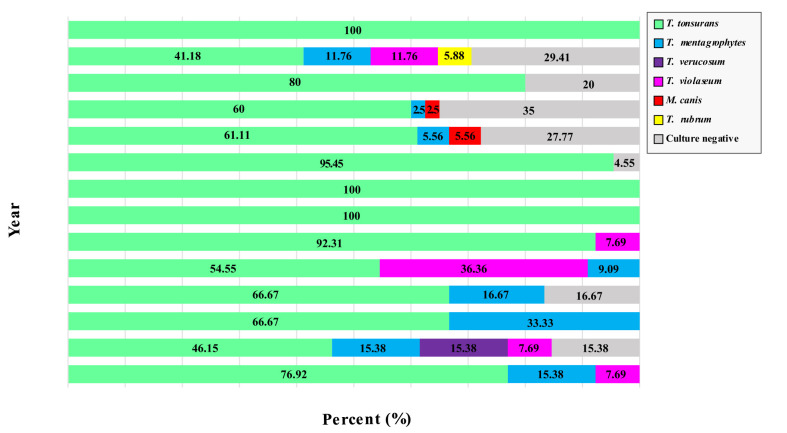
Comparison of the dermatophyte diversity by the year

In general, *T. tonsurans*, as an anthropophilic dermatophyte, was the most common etiological agent (146, 83.91%) among the 174 identified dermatophytes from patients with TC, followed by *T. mentagrophytes* (13, 7.47%), *T. violaceum* (9, 5.17%), *M. canis* (3, 1.71%), *T. verrucosum* (2, 1.15%), and *T. rubrum* (1, 0.57%) (Figure 2b). It is noteworthy that *T. tonsurans* was the most common isolated agent in all age groups. Furthermore, culture-negative results were observed in 66.67% of cases in the age group of 35-39 years old (Figure 2c). Figure 3 shows the diversity of isolated dermatophytes during the year of the study.

## Discussion

Tinea capitis remains a major public health challenge worldwide, particularly in developing countries [ 24 - 27 ]. This retrospective study showed that TC accounts for the infection of 22.7% (209/921) of all suspected patients over a period of 12 years. This rate is higher than that reported by Bassiri Jahromi et al. (13.3%, 209/1568) and lower than that declared by Afshar et al. (27.5%, 480/1745) [ 28 , 29 ]. The prevalence rate of TC varies not only between continents but also from one city to another within the same country depending on the sampling method, the methodology of epidemiological data collection, and the environmental and socioeconomic dynamics of the studied population [ 30 , 31 ]. 

According to a systematic review of worldwide data on TC [ 31 ], the global prevalence rates of TC were 0.4-87.8% in Africa, 1.1-27.5% in South Asia, 0.2-54.0% in Southern Europe, and 2.9-74.0% in America. In the present study, the frequency of TC during the years 2009-2022 showed a fluctuating trend. After a sharp decrease in the prevalence rate in 2016 (6.2%), there was a drastic increase in 2017 (31.8%), followed by a fluctuating pattern until 2020. Since the onset of the pandemic, in early 2020, TC has been on a downward trend (26.32%) due to fear of COVID-19 and avoidance of seeking medical care. COVID-19 led to a great reduction in consultation with a dermatologist and routine care related to skin diseases, including TC. From 2021 to 2022, when fears subsided and vaccination began, the restrictions were lifted and people returned to normal life which led to a gradual increasing trend in TC (37.5%). 

Regarding gender distribution, results of the present study showed that TC was more prevalent among males, compared to females as reported by other authors [ 29 , 32 - 35 ]. The higher frequency of TC in males may be due to their short hair which allows fungal contamination spores to reach the scalp more easily. In addition, males have a higher tendency to play sports, perform outdoor activities, and have poor personal hygiene which increases the chances of exposure to the causative agents [ 34 , 36 ]. It is noteworthy that in the present study, most patients with TC were involved in wrestling (68.9%), a popular sport among males in the Mazandaran province of Iran, which is considered one of the most important transmission routes of TC [ 37 ].

According to the findings, approximately 86% out of 209 patients with TC were under 14 years old. This is similar to the findings of other studies, which reported that TC primarily occurs in children [ 28 , 29 , 32 , 38 , 39 ]. It is believed that the lower incidence rate of TC in adulthood can be due to the fungistatic properties of long-chain fatty acids of sebum as well as the maturation of hair follicles and the immune system at puberty that may protect against fungal invasion [ 36 , 40 ].

Consistent with the results of other studies in Iran, it was found that endothrix was the predominant type of hair invasion [ 29 , 41 ]. In recent years, the incidence rate of tinea favosa has decreased worldwide [ 42 , 43 ] and currently, the infection is almost eradicated in most developed countries [ 44 ]. This is in line with the findings of this study as no evidence of favus was reported.

The predominance of etiological agents in TC varies according to several factors, including geo-graphical areas, climate, age, gender, socio‐economic conditions, hygiene, urbanization, human-animal interaction, ethnicity, overcrowding, migration, lifestyle, environment, general health condition, and passage of the time [ 40 , 45 , 46 ]. Therefore, infectious species may vary from country to country or even within the regions of the same country. Several previous studies have reported *M. canis* as one of the main pathogens of TC in Southern, Eastern and Central Europe, South America, Africa, the Middle East, and Western Asia [ 24 , 47 - 50 ] while *T. violaceum* is one of the most common causative agents of TC in India, China, and Thailand as well as *T. verrucosum* in Central and South Asia [ 31 , 51 - 57 ]. In the present study, the most prevalent dermatophyte isolated from TC was *T. tonsurans* (146/174, 69.9%) followed by *T. mentagrophytes* (13, 6.2%). In contrast to the findings of this research, a study performed in China on children with TC under 18 years old (2011-2019) [ 38 ] reported a shift from anthropophilic to zoophilic dermatophytes in the past two decades. 

In this study, the rate of negative culture results of TC was almost twice the rate of direct microscopic examination which could be due to prior antifungal medications, inadequate specimens, and diseases that mimic TC. Direct microscopic examination and culture are two necessary methods to confirm the TC. In a study conducted by Pai et al., both culture and microscopy positivity were observed in 20/70 (28.6%) patients, and both culture and microscopy negativity were seen in 13/70 (18.5%) patients while 25/70 (35.7%) cases were culture-negative and direct-positive [ 36 ]. In another study, 72 out of 76 patients had at least one positive test while 57 cases had both a positive culture and direct examination. In two cases with both a negative culture and direct examination, seborrheic dermatitis was diagnosed [ 58 ].

As an anthropophilic dermatophyte, *T. tonsurans* was considered a frequent causative agent of TC, especially in athletes of high-contact sports, such as wrestling and judo [ 37 , 59 , 60 ]. In line with the findings of the current study, a previous study on the prevalence of tinea gladiatorum among wrestlers from Mazandaran province reported *T. tonsurans* (192/203, 94.5%) as the most frequent dermatophyte agent followed by *M. canis* (6/203, 2.9%) [ 37 ]. In another study, *T. tonsurans* (186 cases, 38.8%) followed by *T. violaceum* (119 cases, 24.8%) were reported as the predominant causative agents of TC in the northeast of Iran [ 29 ]. 

There is a discrepancy between the predominant agents in this study and some studies conducted in other regions of Iran. *T. mentagrophytes/ interdigitale*, *M. canis*, *T. verrucosum*, and *T. violaceum* were reported as the most common frequent agents in TC in Mashhad [ 46 , 61 ] and Isfahan [ 62 ], Iran, respectively. It is worth to be noted that *T. tonsurans* has recently spread from Latin America to the United States and subsequently, as a major agent of TC in Africa, the Middle East, and other regions [ 63 ]. 

In addition, several studies have also shown that currently, *T. tonsurans*, is the most prevalent cause of TC in the United States, Canada, Mexico, Brazil, Jamaica, the United Kingdom, and parts of Western Europe [ 31 , 51 , 64 ]. The present study has several strengths, including a large sample size that provides more accurate data. Furthermore, the data were obtained from a reference laboratory to which most of the patients suspected of TC were referred and this ensured the validity. 

One of the limitations of the present study was that the species identification was not conducted based on molecular method which may lead to a few discrepancies. However, the classical species identification approaches based on macroscopic and microscopic characteristics are sufficient for the prescription of appropriate medications.

## Conclusion

In this study, it was found that over 80% of TC in Northern Iran is due to the anthropophilic *T. tonsurans* as the causative agent. This is an indication of poor hygiene practices resulting in human-to-human transmission of the agents of dermatophytosis. Therefore, enhanced public health interventions to improve hygiene and improve preventive measures are highly recommended. Despite its prevalence, the absence of appropriate considerations highlights that TC is a neglected complication among children.

## Acknowledgments

Not declared. 

## Ethical Considerations

This study was approved by the Ethics Committee of the Mazandaran University of Medical Sciences, Iran (IR.MAZUMS.REC.1401.392).

## Authors’ contribution

Conceptualization: M.T. H. and T. S., Data curation and resources: F. K., J. J., M. M., M. N., E. S., S. A., E. M., Z. H., M. G., and G. R. R., and A. K. N., Writing-original draft: F. K., M. T. H., and T. S., Writing-review and editing: F. K., M. T. H., F. B, and T. S. All authors read and confirmed the published version of the manuscript.

## Conflicts of interest

The authors have no conflicts of interest to declare.

## Financial disclosure

This study was supported by Mazandaran University of Medical Sciences (Grant number: 1401.14919).
